# Investigating the link between PCP signalling and ciliogenesis during zebrafish development

**DOI:** 10.1186/2046-2530-1-S1-O22

**Published:** 2012-11-16

**Authors:** A Borovina, B Ciruna

**Affiliations:** 1 Hospital for Sick Children, Canada; 2 University of Toronto, Canada

## 

Polycystic kidney disease (PKD) is characterized by dilated collecting tubules causing fluid filled cysts and enlarged kidneys. Dilated tubules can result from disrupted convergence and extension (C&E) movements and defects in oriented cell divisions (OCD). Planar cell polarity (PCP) signalling has been implicated in PKD since defects in C&E and OCD are both characteristic of disrupted PCP. Surprisingly, genetic studies of mutations causing PKD predominantly affect proteins that localize near cilia and basal bodies. The functional relationship between cilia and PCP signalling in the context of PKD is still poorly understood. Previous studies of PCP effector proteins suggested that PCP signalling was required for cilia formation. However, these proteins are not specific to PCP signalling and are shared with other Wnt pathways. To determine the role of a core and specific PCP regulator on ciliogenesis, we examined maternal-zygotic (MZ) *vangl2* zebrafish mutants. Analysis of *MZvangl2* mutants revealed that PCP is not required for cilia formation but is required for the posterior tilting and posterior positioning of motile cilia. To determine whether cilia were required to establish cell polarity, we generated *MZift88* mutants where ciliogenesis is completely abolished. *MZift88* mutants have normal C&E movements, suggesting that cilia are not directly required for PCP mediated morphogenetic movements. However, we have observed defects in PCP-controlled OCD occurring during gastrulation. Remarkably, these divisions occur prior to cilia formation, suggesting a cilia-independent role for IFT proteins in cell divisions. We are currently determining whether IFT88 regulates all OCD or just those that are PCP-controlled.

